# Detection of cell-free histones in the cerebrospinal fluid of pediatric central nervous system malignancies by imaging flow cytometry

**DOI:** 10.3389/fmolb.2023.1254699

**Published:** 2023-11-01

**Authors:** Diana Buzova, Jan Frohlich, Danica Zapletalova, Marco Raffaele, Oriana Lo Re, Desislava K. Tsoneva, Jaroslav Sterba, Jan Cerveny, Manlio Vinciguerra

**Affiliations:** ^1^ Department of Adaptive Biotechnologies, Global Change Research Institute CAS, Brno, Czechia; ^2^ International Clinical Research Center, St Anne’s University Hospital, Brno, Czechia; ^3^ Department of Pediatric Oncology, University Hospital Brno and Faculty of Medicine, Masaryk University, Brno, Czechia; ^4^ Department of Stem Cell Biology and Transplantology, Research Institute of the Medical University of Varna, Varna, Bulgaria; ^5^ Department of Medical Genetics, Medical University of Varna, Varna, Bulgaria; ^6^ Faculty of Health, Liverpool John Moores University, Liverpool, United Kingdom

**Keywords:** histones, pediatric brain tumors, liquid biopsy, epigenetics, imaging

## Abstract

**Introduction:** Pediatric brain tumours (PBT) are one of the most common malignancies during childhood, with variable severity according to the location and histological type. Certain types of gliomas, such a glioblastoma and diffuse intrinsic pontine glioma (DIPG), have a much higher mortality than ependymoma and medulloblastoma. Early detection of PBT is essential for diagnosis and therapeutic interventions. Liquid biopsies have been demonstrated using cerebrospinal fluid (CSF), mostly restricted to cell free DNA, which display limitations of quantity and integrity. In this pilot study, we sought to demonstrate the detectability and robustness of cell free histones in the CSF.

**Methods:** We collected CSF samples from a pilot cohort of 8 children with brain tumours including DIPG, medulloblastoma, glioblastoma, ependymoma and others. As controls, we collected CSF samples from nine children with unrelated blood malignancies and without brain tumours. We applied a multichannel flow imaging approach on ImageStream(X) to image indiviual histone or histone complexes on different channels.

**Results:** Single histones (H2A, macroH2A1.1, macroH2A1.2 H2B, H3, H4 and histone H3 bearing the H3K27M mutation), and histone complexes are specifically detectable in the CSF of PBT patients. H2A and its variants macroH2A1.1/macroH2A1/2 displayed the strongest signal and abundance, together with disease associated H3K27M. In contrast, mostly H4 is detectable in the CSF of pediatric patients with blood malignancies.

**Discussion:** In conclusion, free histones and histone complexes are detectable with a strong signal in the CSF of children affected by brain tumours, using ImageStream(X) technology and may provide additive diagnostic and predictive information.

## 1 Introduction

Pediatric brain tumours (PBT) are the most frequent malignancy, after leukaemia, during childhood and are the chief cause of pediatric cancer-related morbidity and mortality (Jessa et al., 2019). A world incidence of approximately 3–5 cases per 100,000 live births represents about 20% of all cancers in children (Baade et al., 2010; Johnson et al., 2014). Among them medulloblastoma, high-grade gliomas (HGGs) that include diffuse intrinsic pontine glioma (DIPG) and ependymoma, account for approximately 20%, 12% and 8% of all PBT, respectively (Hargrave and Zacharoulis, 2007). While tumour resection for some PBT (e.g., low-grade gliomas) are potentially both diagnostic and curative, many other PBT (e.g., DIPG) are not eligible for surgical removal, because of their infiltrating nature or their neuroanatomical location.

Until 2016, the classification of brain tumours was based mainly on histology, where tumours were ranked according to their common morphologic features with distinct attributed cells of origin and according to different stages of development. Subsequently, the WHO incorporated molecular findings into the diagnosis of brain tumours (Louis et al., 2016; Miller, 2023). For example, medulloblastoma can be now classified as different histological-molecular combinations of 4 histological variants and newly defined 4 molecular subgroups (Taylor et al., 2012). Several large projects have started to profile the genetic background of both adult and pediatric central nervous system (CNS) tumours. Genetic divergences between pediatric and adult tumours could explain some of the diverse responses to chemotherapy (Paugh et al., 2010). For example, H3K27M mutation has been detected in approximately 80% of DIPG patients, primarily occurring in children, while IDH1 and IDH2 mutations that characterize adult glioma are generally absent. Moreover, hypermethylation of RASSF1A, HIC1 and CDH1 was detected in pediatric medulloblastoma (Sexton-Oates et al., 2015). In the long term, the identification and monitoring of molecular changes would likely be critical for the clinical management of pediatric tumours, overcoming the limitations of magnetic resonance imaging (MRI) and of histology (Northcott et al., 2017; Salloum et al., 2017).

In this regard, liquid biopsies are emerging as a promising platform for cancer (Tsoneva et al., 2023; Tsoneva et al., 2023). Biofluids, such as blood and cerebrospinal fluid (CSF), may include small amounts of circulating tumour cells, cell free DNA (cfDNA), fragmented peptides and intact proteins. To be suitable as clinically useful biomarkers, they must be highly specific to the tumour and detectable. High levels of cell free tumour DNA (ctDNA) in plasma of adult and pediatric patients with advanced tumours have been associated with dismal prognosis (Mehrotra et al., 2018; Andersson et al., 2020; Liu et al., 2022). However, because of the blood-brain barrier, in the case of brain tumours plasma contains significantly lower amounts of ctDNA, while CSF is in intimate contact with brain malignancies and may represent a better source of ctDNA (Seoane et al., 2019; McEwen et al., 2020). CSF ctDNA analyses have been aimed toward the detection of PBT-associated mutations (Sun et al., 2021). For instance, H3K27M mutation is associated with a poorer clinical outcome (Lu et al., 2018; Panditharatna et al., 2018). Nevertheless, PBT have shown lower incidence of mutations when compared to adult brain tumours (Parsons et al., 2011), making their detection difficult. Moreover, methods of processing liquid biopsy samples have not been fully developed and standardized yet and ctDNA integrity is regulated by factors such as temperature and storage methods (Parpart-Li et al., 2017; Li et al., 2021). On the other hand, proteins derived from tumour cells are also secreted into the plasma and the CSF, representing a more robust and detectable alternative for the diagnosis and monitoring of malignancies and neurological disorders (Saratsis et al., 2012; Humphries et al., 2014; Di Meo et al., 2017; Schilde et al., 2018).

Histones are highly basic proteins organized in an octameric core around DNA wrapped to form the nucleosome, the repeating unit of chromatin (Thomas and Kornberg, 1975). Elevated nucleosome levels were detected and used as diagnostic tool in several adult cancers and in obesity (Holdenrieder et al., 2004; Bauden et al., 2015; Rahier et al., 2017; Lo Re et al., 2019). Besides the “canonical” histones, there were described 19 variants of H2A and 6 variants of H3, in human somatic cells (Buschbeck and Hake, 2017). The differences among the variants are related to their particular temporal pattern of incorporation into the chromatin during the cell cycle (Buschbeck and Hake, 2017). The variants macroH2A1 and macroH2A2 are the largest among the histone proteins (Buschbeck and Hake, 2017). Others and we have shown that macroH2A1 isoforms play a pivotal role in regulating stem cell differentiation and cell plasticity (Pazienza et al., 2014; Borghesan et al., 2016; Lo Re et al., 2018a; Lo Re et al., 2018b; Bereshchenko et al., 2019; Lo Re et al., 2020). It remains to be elucidated whether nucleosomes or distinct circulating histones patterns may be employed as new biomarkers for PBT. High resolution ImageStream(X) imaging flow cytometer has been used to detect and quantify in a multiplex fashion the expression levels of biomarkers on circulating blood and cancer cells, with high speed, reliability, and cheaply (Ogle et al., 2016). We recently developed an ImageStream(X)-based method to identify a circulating histone signature able to discriminate between pediatric and adult patients affected by non-alcoholic fatty liver disease (Buzova et al., 2020; Buzova et al., 2022). In this study, we developed an ImageStream(X)-based method to detect circulating histones in the CSF of patients with PBT, providing a new potential tool applicable in PBT diagnosis and prognosis.

## 2 Results

### 2.1 Circulating histones are detectable in the cerebrospinal fluid (CSF) of children with CNS and hematological tumours

**FIGURE 1:**
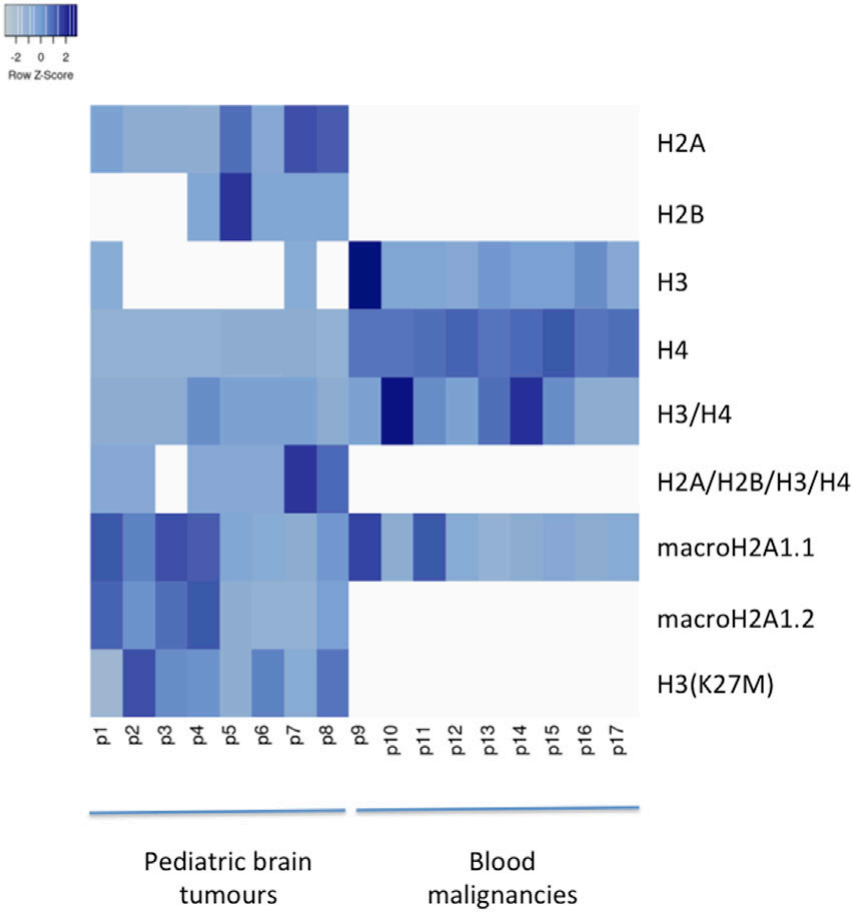
Heatmap of histone frequencies (H2A, H2B, H3, H4, H3/H4, H2A/H2B/H3/H4, macroH2A1.1, macroH2A1.2, H3(K27M) characterizing each CSF sample from PBT patients (from 1 to 8) and from patients with blood malignancies (from 9 to 17). Higher frequencies are indicated with darker shades of blue. Generated with Heatmapper (Babicki et al., 2016).

**FIGURE 2:**
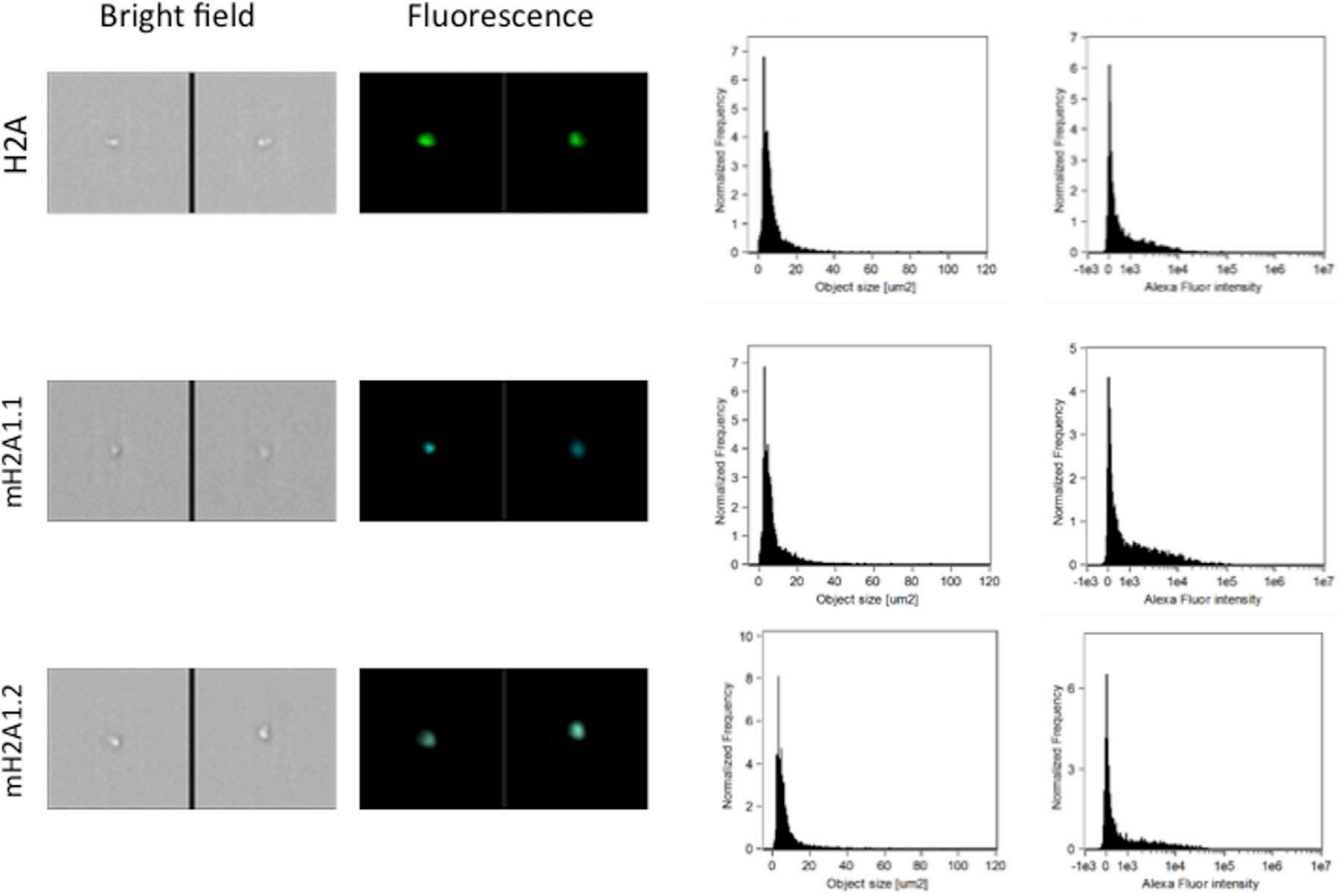
Representative images, size distribution and fluorescence signal intensity from fluorescence marker of histones H2A, two large variants of histone H2A (macroH2A1.1, macroH2A1.2) and histone H3K27M. ImageStream photographs show bright-field images and histone staining (fluorescence from Alexa Fluor^®^ 488).

**FIGURE 3:**
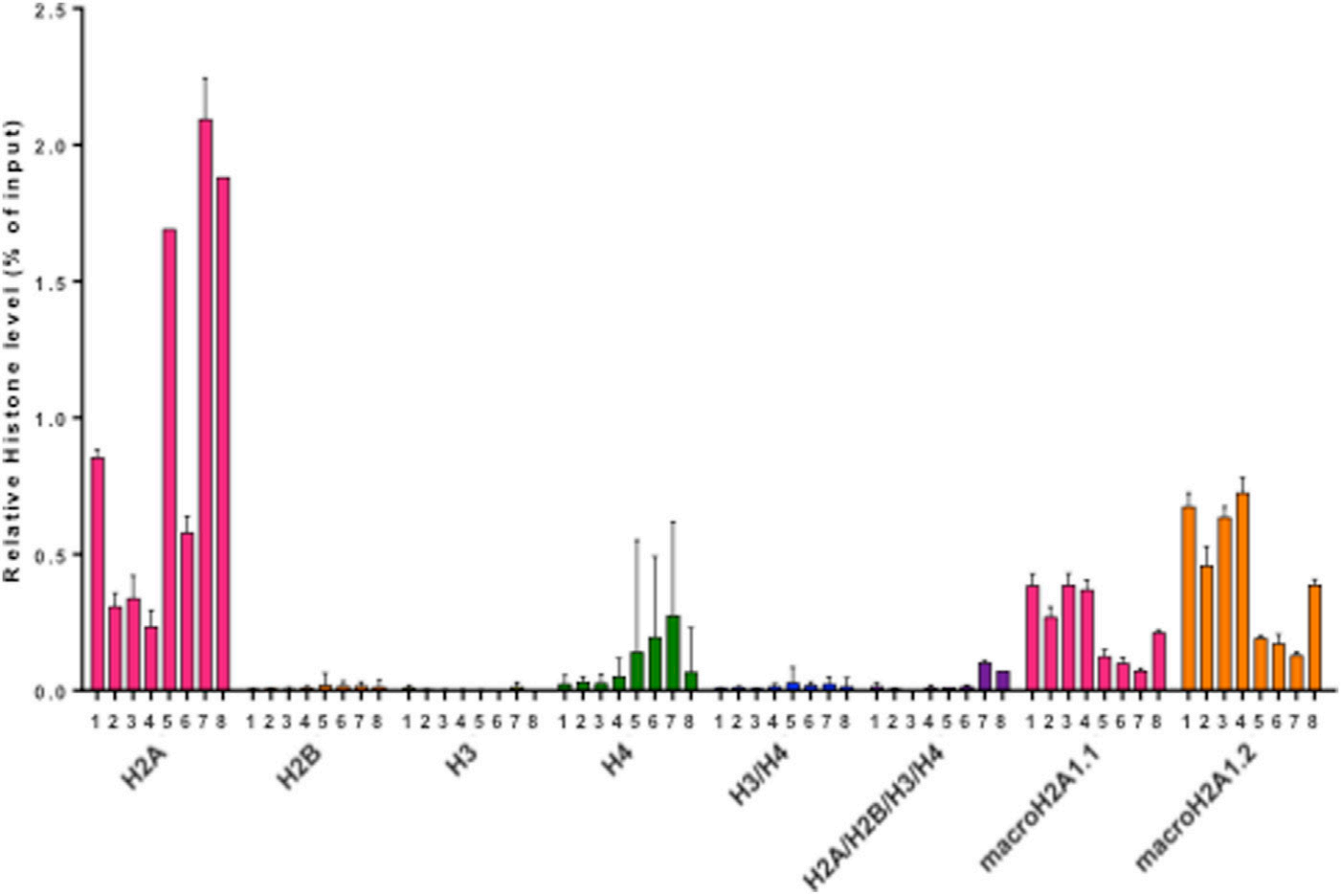
Histogram representing relative abundances of histone species (H2A, H2B, H3, H4, H3/H4, H2A/H2B/H3/H4, macroH2A1.1, macroH2A1.2, H3(K27M), expressed as % of input. Different colors represent individual histone species/complexes detected in the eight CSF samples from PBT patients.

Pediatric CNS tumours need minimally invasive molecular profiling, in order to monitor tumour response and progression. Therefore, we sought to determine if individual histones (H2A, macroH2A1.1, macroH2A1.2, H2B, H3, H4 and histone H3 bearing the H3K27M mutation), and histone complexes might be detectable in the CSF of PBT patients. ELISA tests can identify nucleosome or single histones in biofluids; nevertheless, a high throughput real-time monitoring of multiple histones is lacking. In this study, we have optimized a protocol that we have previously developed (Buzova et al., 2020), in order to develop a multi-channel flow imaging methodology on ImageStream(X) and image individual histone staining on different wavelengths/channels. We analyzed the four canonical histones (H2A, H2B, H3, H4), two variants of histone H2A (macroH2A1.1 and macroH2A1.2) and the H3K27M mutated H3 histone, in the CSF of the eight patients with PBT and nine patients affected by unrelated blood malignancies (without PBT) (Table 1). Landmarks studies have demonstrated that histone dimers may provide stable intermediate during nucleosome assembly, and trimers do not form (Sperling and Bustin, 1976). Individual histone types can be considered as an interchangeable subunit of a larger complex where the dimer species is the most stable sub-complex (Sperling and Bustin, 1976). In this respect, histones assemble H2A-H2B heterodimers and H3-H4 heterotetramers in a preferential manner (Luger et al., 1997). Subsequently, in presence of DNA, H2A/H2B dimer binds to the H3/H4 tetramer because of interactions between H2B and H4 (Luger et al., 1997). In light of this, we probed the above-mentioned seven individual histones (H2A, H2B, macroH2A1.1, macroH2A1.2, H3, H4 and the H3K27M mutated histone) together with the dimers H3/H4, and the heterotetramers H2A/H2B/H3/H4. We detected all histone species, variants and complexes at different frequencies in the CSF of PBT patients (Figure 1, left panel). By contrast, only H3, H4 and macroH2A1.1 were detectable in the CSF of patients affected by blood malignancies, which displayed higher frequencies of H4 levels (Figure 1, right panel). Figure 2 exemplifies, using *ad hoc* primary and secondary antibodies, the single imaging of histones H2A, macroH2A1.1, macroH2A1.2 and H3K27M in four different imaging channels in the CSF of PBT patients. Interestingly, we observed a generally higher abundance of histones H2A, macroH2A1.1, macroH2A1.2 and H3 bearing the K27M mutation compared to H2B, H3, H4 and the histones complex (H3/H4; H2A/H2B/H3/H4) in each sample (Figure 3). Also, there was an evident opposite trend between the abundance of H2A and macroH2A1.1/macroH2A1.2 levels in the CSF samples (Figure 3). Due to the limited number of samples in this pilot study, a comparative and statistically significant analysis between PBT types and/or disease stage was not possible. In summary, we detected for the first-time circulating histones in CSF of pediatric CNS tumours patients, and in CSF in general, using a non-invasive and fast ImageStream (X)-based imaging method. These pilot findings offer a proof-of-concept of new histone-based liquid biopsies, which may enable tumour epigenetic characterization by minimally invasive means.

**TABLE 1:** Patient data. 1–8: disease characteristics and demographics of pediatric patients with brain tumors (PBT). 9–18: disease characteristics and demographics of pediatric patients without PBT and with unrelated blood malignancies. LP, lumbar puncture.

Patient	Gender	Age at the time of diagnosis	Diagnosis	Localization	Localised vs. metastatic	Treatment before LP	Intrathecal therapy before LP	Condition at the tie of lumbar puncture	Malignant cells in CSF
1	M	19	Diffuse midline glioma with histone H3F3A K27M mutation	Intraspinal intramedullar tumor of conus medullaris till Th11-12	Metastases in cerebellar hemispheres	Sine	No	Staging before treatment	No
2	M	8	RELA+ anaplastic ependymoma	Supratentorial frontotemporal region	Localised	Surgery, radiotherapy	No	Complete response after surgery and radiotherapy	No
3	M	2	Medulloblastoma, classic, molec.subgroup D	fossa posterior	Metastases in spine Th7/8, malignant cells in CSF	Surgery, chemotherapy (VCR, VP-16, CPM, CDDP, MTX, thiotepa, carboplatin)	No	Partial response after high-dose chemotherapy, metastasis in spine	No
4	M	11	Non-germinatous germ cell tumor of CNS	Regio pinealis	Metastases in spine C1 and Th1	Chemotherapy (carboplatin, VP-16, IFO, CDDP, VBL)	No	Progression of the tumor after chemotherapy, metastases in spine	Yes
5	M	10	Medulloblastoma, classic, molec.subgroup SHH	Fossa posterior	Initially localised, 05/2015 combined metastatic relapse - leptomeningeal and intraspinal metastases throughout whole spine and meninges in fossa posterior	Surgery, radiotherapy, chemotherapy (VCR, CCNU, CDDP), Avastin	Yes (VP-16, MTX, Depocyte)	Progression of metastases in spine, malignant cells in CSF	Yes
6	M	9	Ependymoma grade 2	Fossa posterior	Localised	Surgery	No	Partial response after surgery	No
7	M	5	Medulloblastoma, classic, molec.subgroup D	Fossa posterior	Initially localised, 05/2016 metastatic relapse with ependymal metastases	Surgery, radiotherapy, chemotherapy (Temozolomide, VCR, CDDP, VP-16, CPM), Avastin	Yes (VP-16, Depocyte)	Progression of metastases in CNS	No
8	F	0	Initially diagnosed as PNET, 02/2017 relapse - epitheliolid glioblastoma	Fronto-temporoparietal region	Initially localised, 02/2017 metastatic relapse with meningeal metastases	Surgery, chemotherapy (VECC, Thio, Carbo)	Yes (VP-16, Depocyte)	Relapse	No
9	M	1	PreB/partial proB ALL (MLL+)	Blood/systemic	Blood/systemic	None	NA	Diagnosis	No
10	M	16	intermediate T-ALL	Blood/systemic	Blood/systemic	None	NA	Diagnosis	No
11	F	3	cALL	Blood/systemic	Blood/systemic	None	NA	Diagnosis	No
12	F	2	cALL	Blood/systemic	Blood/systemic	None	NA	Diagnosis	No
13	M	5	cortical T-ALL	Blood/systemic	Blood/systemic	None	NA	Diagnosis	No
14	M	6	preB ALL (E2A/PBX1	Blood/systemic	Blood/systemic	None	NA	Diagnosis	No
			positive)						
15	F	4	cALL	Blood/systemic	Blood/systemic	None	NA	Diagnosis	No
16	F	12	preB ALL	Blood/systemic	Blood/systemic	None	NA	Diagnosis	No
17	M	7	cALL	Blood/systemic	Blood/systemic	None	NA	Diagnosis	No

## 3 Discussion

In this report we show for the first time, to the best of our knowledge, that individual histones and histone complexes can be detected with a strong signal in the CSF of children affected by brain tumours, using ImageStream(X) technology combining flow cytometry with high content image analysis. The advantage ImageStream(X) over conventional flow cytometry [where only forward scatter (FSC) and side scatter (SSC) measures are used to assess cell volume and morphological complexity] is that measurements obtained from an image (called “features”) are up to 86 object-based measurements like size, shape, intensity, circularity (Dominical et al., 2017; Buzova et al., 2020; Buzova et al., 2022), which can capture the complexity of circulating histones and histone complexes. Moreover, the rich multivariate dataset derived from the large numbers of image features, with the assistance of machine learning, could offer clear advantages in future automated cell-free histone image analysis, enabling high-throughput identification as it was demonstrated for other biological entities (Demagny et al., 2022; Rees et al., 2022).

While it has been established that H3K27M gene mutation correlates with a poorer clinical outcome in PBT (Lu et al., 2018; Panditharatna et al., 2018), ours is the first report to show the expression of H3K27M histone protein in the CSF, which is likely to derive from dying tumour cells.

Pediatric brain tumours represent the most common solid malignancy in childhood. Despite multimodal intensive therapies many PBT have poor prognosis and the long-lasting effects of these therapies are very often devastating (Jessa et al., 2019). The inclusion of molecular findings into the classification of brain tumours can help elucidating the biological nature of tumour tissue and therefore can help set up more specific treatment modalities based on personalized medicine. Determining the molecular details of a particular tumour thus requires a sample of tumour tissue by surgery or at least a biopsy. However, some PBT [e.g., diffuse midline glioma, DMG] are not suitable for surgical removal, because of their infiltrating growth or their location, which is not accessible for resection and where even a biopsy of tumour tissue may be associated with life-threating complications. The problem is not only the precise diagnosis of CNS tumours, but also the monitoring of molecular-genetic changes within the tumour tissue during treatment. The monitoring of the treatment response itself is sometimes problematic as the surgery or biopsy is usually performed at the diagnosis or in the case of relapse. Therefore, there is an urgent need for a reliable biomarker in pediatric oncology that could provide more detailed information on diagnosis, tumour classification, disease extent, risk assessment, or to monitor tumour response to treatment, or help identify potential treatment targets, from better available biological material. In CNS tumours, as well as for the diseases and injuried of the spinal cord, CSF is such an optimal medium (Bonner et al., 2018; Siddiq et al., 2021). Several studies demonstrated that it is possible to isolate circulating tumour DNA (ctDNA), extracellular vesicles (EV), and tumour-specific peptides from liquid biopsies (Bounajem et al., 2020).

Liquid biopsy has been studied to obtain material for molecular-genetic testing in a more accessible and less invasive way (Gojo et al., 2019; Azad et al., 2020). Circulating tumour DNA has been successfully isolated from CSF of patients with DMG with H3K27M mutation and was correlated with tumour progression on MRI (Panditharatna et al., 2018). Also, the correlation between levels of ctDNA and treatment has been demonstrated *in vitro* (Stallard et al., 2018). To our best knowledge, no studies so far looked at the presence of histones in cerebrospinal fluid in children with a tumour of CNS.

The variability in histone composition and post-translational modifications within nucleosomes provide a vast and promising diagnostic and prognostic potential. However, whether circulating individual histones or histone complexes play distinct roles as cancer biomarkers is a matter of investigation. Histones can be released from neutrophils (Brinkmann et al., 2004) by extracellular traps. Neutrophil extracellular traps (NETs) are complexes of extracellular fibers made of neutrophil genomic DNA, core histones and other factors. Inflammation-driven increased release of NETs may lead to a characteristic fashion of immune cell death named “NETosis” which causes histone release. Whereas NETs are observed in the CSF in traumatic and infectious CNS conditions (Manda-Handzlik and Demkow, 2019), their role in PBT has not been analyzed. Moreover, together with NETosis-mediated histone release, tumour cells can release histones upon apoptosis as well. When apoptosis occurs, histones might part ways from genomic DNA, which leads to the subsequent release outside the cells and in DNA fragmentation (Wu et al., 2002). This is a pilot study with a limited sample size, whose aim was to establish a proof-of-concept for the detection of cell free histones in the CSF of eight children affected by PBT and, for this reason, did not allow us to compare conditions or to consider covariates. In fact, the impact of therapy and other factors influencing the clinical course of PBT on circulating histone complex composition is not yet understood: a larger sample size is needed to determine whether there is a nexus between circulating histones and PBT. Studies on the liquid biome (cfDNA or histones) might redefine our approach to managing childhood CNS tumors as well as our understanding of the associated molecular landscape.

## 4 Materials and methods

### 4.1 Patients

We assembled a pilot cohort of eight children with pediatric brain tumours including DMG, medulloblastoma, glioblastoma, ependymoma or non-germinoma germ cell tumors (NGGCT); together with nine children with hematological malignancies but without brain tumours; from the Pediatric Oncology Department of Brno University Hospital, of whose CSF samples were available. CSF samples were collected by lumbar puncture for routine diagnostic or prognostic purposes in the period from March 2017 and April 2018. The local ethics committee of the Masaryk University Brno reviewed the studies involving human participants. Participants’ legal guardian/next of kin provided written informed consent to participate in this study. After the laboratory examination residual CSF samples were centrifuged to remove cells and the supernatant was stored at −80°C.

### 4.2 ImageStream(X) protocol optimization

To measure histones we used multispectral imaging flow cytometer ImageStream MkII (Luminex Corporation). During the experimental setup, first we included each sample stained with a single antibody; subsequently, we regulated the power of the appropriate laser not to indclude saturated pixels. We employed the features “Raw Max Pixel” and feature “Saturation Count” [which can be assessed in the IDEAS^®^ statistical analysis software package (Amnis Corporation, United States)], indicating reports the number of saturated pixels in the images. Pixel intensities are reported on the camera pixels from 0 to 4,095 (12 bit) and hence become saturated and cannot be quantified for values greater than 4,095. Individual color controls were employed to extrapolate a spectral crosstalk matrix that was applied to the image files to assign probed images to individual imaging channels. The outcome, compensated image files, were processed and results were analyzed thanks to image-based algorithms included in the IDEAS^®^ statistical analysis software package. During the measurements, we started from higher voltage to lower. Identifying optimal laser power for each individual antibody (laser 488 nm–5 mW, laser 561 nm–20 mW, laser 642–5 mW) was adopted for the multichannel test.

Single-color controls were used to calculate a spectral crosstalk matrix that was applied to the image files in order to isolate probed images to single imaging channels. The resulting compensated image files were analyzed using image-based algorithms available in the IDEAS^®^ statistical analysis software package (Amnis Corporation, United States) and analysis of the results was done with the same software.

### 4.3 ImageStream(X) detection of histone complexes in the cerebrospinal fluid of pediatric patients with CNS tumours

We employed four staining mixes: three including four different primary antibodies and four species-matching secondary antibodies and one including three different primary antibodies and three species-matching secondary antibodies.

First staining set. Primary antibodies: anti-macroH2A1.1 (Cell Signaling Technology, 12455S, United States), anti-histone H2B (Abcam, Ab134211, United States), anti-histone H4 (Abcam, Ab31830, United States), anti-histone H3 (Abcam, Ab12079, United States). Secondary antibodies: anti-rabbit IgG H&L-AlexaFluor^®^ 488 (Thermo Fisher Scientific, A-11008, United States), anti-chicken IgY H&L-Alexa Fluor^®^ 594 (Thermo Fisher Scientific, A-11042, United States), anti-mouse IgG H&L-Alexa Fluor^®^ 647 (Thermo Fisher Scientific, A-21235, United States); anti-goat IgG H&L Alexa Fluor^®^ 555 (Thermo Fisher Scientific, A-21432, United States).

Second set. Primary antibodies: anti-macroH2A1.2 (Cell Signaling Technology, 4827S, United States), anti-histone H2B (Abcam, Ab134211, United States), anti-histone H4 (Abcam, Ab31830, United States); anti-histone H3 (Abcam, Ab12079, United States) anti-histone H3. Secondary antibodies: anti-rabbit IgG H&L-Alexa Fluor^®^ 488 (Thermo Fisher Scientific, A-11008, United States), anti-chicken IgY H&L-Alexa Fluor^®^ 594 (Thermo Fisher Scientific, A-11042, United States), anti-mouse IgG H&L-Alexa Fluor^®^ 647 (Thermo Fisher Scientific, A-21235, United States); anti-goat IgG H&L Alexa Fluor^®^ 555 (Thermo Fisher Scientific, A-21432, United States).

Third set. Primary antibodies: anti-histone H2A (Abcam, Ab18255, United States), anti-histone H2B (Abcam, Ab134211, United States), anti-histone H4 (Abcam, Ab31830, United States), anti-histone H3 (Abcam, Ab12079, United States). Secondary antibodies: anti-rabbit IgG H&L-Alexa Fluor^®^ 488 (Thermo Fisher Scientific, A-11008, United States), anti-chicken IgY H&L-Alexa Fluor^®^ 594 (Thermo Fisher Scientific, A-11042, United States), anti-mouse IgG H&L-Alexa Fluor^®^ 647 (Thermo Fisher Scientific, A-21235, United States); anti-goat IgG H&L Alexa Fluor^®^ 555 (Thermo Fisher Scientific, A-21432, United States).

Fourth set. Primary antibodies: histone H3 (K27M Mutant Specific) (D3B5T) Rabbit mAb (Cell Signaling Technology, 74829S, United States), anti-histone H2B (Abcam, Ab134211, United States), anti-histone H4 (Abcam, Ab31830, United States). Secondary antibodies: anti-rabbit IgG H&L-Alexa Fluor^®^ 488 (Thermo Fisher Scientific, A-11008, United States), anti-chicken IgY H&L-Alexa Fluor^®^ 594 (Thermo Fisher Scientific, A-11042, United States), anti-mouse IgG H&L-Alexa Fluor^®^ 647 (Thermo Fisher Scientific, A-21235, United States).

The choice of the different primary antibodies in the mixes relied on preliminary testing of available flow cytometry-grade commercially antibodies; while the choice of species-matching secondary antibodies relied on the possibility to use simultaneously fluorophores (excitable at 488, 555, 594 or 647) that could be detectable on individual ImageStream channels.

Sample preparation: for each individual CSF sample from PBT patients, 50 μL were incubated overnight at 4°C with four (or three, according to the staining set reported above) primary antibodies from each set, always in a ratio of 1:50. The phosphate buffer (pH 7.4) was used to dilute them, and the antibodies were added consecutively from separate solutions. The following day, the sample was incubated for 2 h at room temperature with four (or three, according to the staining set reported above) fluorescent secondary antibodies from each set, in a ratio of 1: 100 (the same ratio for each secondary antibody, for which dilution was used phosphate buffer—pH 7.4 and the antibodies were added from separate solutions, one after the other), for 2 h at RT.

For every stained CSF sample, 10,000 objects were measured employing an excitation laser 488 nm (5 mW) for Alexa Fluor^®^ 488 and fluorescence was detected in channel two (505–560 nm), 561 nm (20 mW) for Alexa Fluor^®^ 555 and Alexa Fluor^®^ 594 and fluorescence was detected in channel three (560–595 nm) and channel four (595–642 nm), 642 nm (5 mW) for Alexa Fluor^®^ 647 and fluorescence was detected in channel five (642–745 nm). The bright field image was instead collected in channel one and the laser scatter image in channel six.

To measure fluorescence-stained objects within all measured objects, gating was applied to discern: 1) focused objects and 2) fluorescent objects. The image files were subsequently processed and analyzed using image-based algorithms available in the IDEAS^®^ statistical analysis software package.

### 4.4 Statistical analyses

All statistical tests were performed using GraphPad Prism (version 6.0, GraphPad Software, United States) or SPSS Statistics software (version 22.0, IBM Corporation, United States). As a first step, the Kolmogorov-Smirnov test was used to assess the normal distribution of continuous variables prior to further analyses. Categorical variables were compared using the Chi-squared test. Continuous variables with normal distribution were instead compared using Student’s *t*-test. Continuous variables underlying a skewed distribution were compared using the Mann-Whitney U or Kruskal–Wallis tests. All statistical tests used were two-sided, and *p* values < 0.05 were considered statistically significant.

## Data Availability

The raw data supporting the conclusion of this article will be made available by the authors, without undue reservation.
